# Invasive Pneumococcal Disease in Latvia in PCV10 Vaccination Era, 2012–2018

**DOI:** 10.3389/fped.2021.532489

**Published:** 2021-10-07

**Authors:** Larisa Savrasova, Angelika Krumina, Hedija Cupeca, Indra Zeltina, Anita Villerusha, Ilze Grope, Ludmila Viksna, Elina Dimina, Sooria Balasegaram

**Affiliations:** ^1^Centre for Disease Prevention and Control of Latvia, The European Programme for Intervention Epidemiology Training (EPIET), Riga Stradinš University, Riga, Latvia; ^2^Department of Infectology, Riga Stradinš University, Riga, Latvia; ^3^Department of Pediatrics, Riga Stradinš University, Riga, Latvia; ^4^Department of Public Health and Epidemiology, Riga Stradinš University, Riga, Latvia; ^5^Centre for Disease Prevention and Control of Latvia, Riga, Latvia; ^6^The European Programme for Intervention Epidemiology Training (EPIET) Coordinator, Public Health England Field Epidemiology Service South East and London, London, United Kingdom

**Keywords:** invasive pneumococcal disease, pneumococcal conjugate vaccine (PCV), *S. pneumoniae* serotype replacement, Latvia, PCV10

## Abstract

In 2010 in Latvia, invasive pneumococcal disease (IPD) became a cause for concern and vaccination of infants with four doses of 7–valent pneumococcal conjugate vaccine (PCV7) commenced. In 2012, 10–valent pneumococcal conjugate vaccine (PCV10) (three doses at 2, 4, and 12–15 month of age) vaccination was introduced. We described incidence and serotype distribution of IPD in Latvia and investigated serotypes associated with death from IPD based on surveillance data. Adult vaccination against pneumococcal infection is not included in the national immunization program. Laboratory confirmed IPD cases are passively notified to the Center for Disease Prevention and Control of Latvia (CDPC) by laboratories and clinicians. We calculated incidence by age, sex, case fatality, and trend in serotypes by conducting a retrospective population-based cross-sectional study based on national IPD surveillance data. From 2012 to 2018 466 cases of IPD were reported. The highest notified incidence was in 2015 at 4.4/100,000, which fell to 3.9 in 2018. The highest mean annual IPD incidence was in infants (4.8) and in the elderly (6.0). PCV10 vaccine serotypes were the most prevalent in IPD cases up to 2015 with a decreasing trend from 50% (20/40) in 2012 to 19% (14/74) in 2018 (chi2 test for trend of odds = 0.000). PCV23nonPCV13 vaccine serotypes had an increasing trend and rose from 18% (7/40) to 34% (25/74) (chi2 test for trend of odds = 0.000). Non-Vaccine serotypes had an increasing trend and rose from 13% (5/40) to 27% (20/74) (chi2 test for trend of odds = 0.038). Reported total case fatality was 19% (87/466). The highest, at 36% (20/56), was reported in 2013. After adjusting for age, *Streptococcus pneumoniae* serotype 3 was associated with death from IPD (adjusted OR 2.3 95%CI 1.25–4.12 p 0.007). Surveillance data indicate evidence of serotype replacement with an increasing trend of serotype 19A and PPV23nonPCV13 and Non-Vaccine serotypes. Serotype 3 and age were associated with fatal IPD outcome. Further studies of *S. pneumoniae* carriage would be useful in providing more evidence to characterize serotypes' circulation.

## Introduction

Invasive pneumococcal disease (IPD) is an acute and life-threatening disease caused by *Streptococcus pneumoniae* (*S. pneumoniae*), a common commensal of the upper respiratory tract (1). The invasive disease encompasses a severe range of syndromes, including meningitis and bacteremia, and may result in serious sequelae and permanent impairment. Children are at major risk, as are immunocompromised patients and the elderly. The World Health Organization estimates that 476,000 deaths among children <5 years in 2008 were caused by pneumococcal infection (2).

The introduction of vaccines to prevent pneumococcal disease has proven to be very effective in reducing the incidence of IPD (2, 3). In Europe prior to immunization with PCV7, the mean annual incidence of IPD in children aged <2 was 44.4 per 100 000 population (2). PCV7 was first introduced into the National Immunization program in Luxemburg (2003), followed by Germany, Greece, Italy, Liechtenstein, the Netherlands, Norway, Switzerland, and the UK in 2006 (4). Many European countries demonstrated a decline in IPD cases after PCV7 introduction (5–7). The most common serotypes causing IPD in Europe prior to PCV7 introduction were 14, 6B, 19F, and 23F and post-introduction were 1, 19A, 3, 6A, and 7F (4). Two PCV vaccines (10-valent and 13-valent) have been available since 2009. Germany first introduced PCV10 and PCV13 in 2009, followed by several European countries (8, 9). Central and Eastern Europe PCV vaccination was introduced from 2007 in Croatia, 2008 in Hungary and Turkey (PCV7), 2009 in Slovakia and Poland (PCV7), 2010 in the Czech Republic (PCV7) and Kazakhstan (PCV13), and in 2014 in Russia (PCV13) (10). Since PCV introduction into countries' immunization programs, serotype replacement of *S. pneumoniae* has been noticed, despite overall IPD incidence decreasing (11). The proportion of overall IPD attributed to serotypes in the vaccine has decreased and the proportion attributed to Non-Vaccine serotypes increased in high valent PCV era in both <5 (11, 12) and ≥65 (3, 11) age groups in a majority of countries.

In Latvia, invasive pneumococcal disease becomes a cause for concern in 2009 (13). Subsequently, Latvia developed a laboratory and clinical IPD surveillance system based on clinicians and laboratory reports to the CDPC. The system's aims are characterization of local trends, detection of geographic and temporal changes in the prevalence of drug resistant *S. pneumoniae*, monitoring the impact of vaccines on disease, and serotype replacement monitoring.

Latvia introduced a vaccination against pneumococcal disease in 2010 (PCV7), but in 2012 changed the vaccine to PCV10. This is the vaccine which is still used, is free of charge, and recommended to all children. Now immunization against pneumococcal disease consists of 2 + 1 doses: at 2, 4, and 12–15 months. While PCV vaccination is offered to all infants in Latvia and is a part of the National Immunization program, adult vaccination against pneumococcal infection is not free of charge and recommendations for adult vaccination only began in 2019.

The objectives of our study were to describe the incidence trend and serotype distribution trend of IPD in Latvia and to investigate serotypes associated with death from IPD based on surveillance data.

## Materials and Methods

### Study Population and Design

We conducted a retrospective population-based cross-sectional study based on national IPD surveillance data over a 7-year period (2012–2018).

Latvia is a small country with an average population of 1,987,093 (14). Riga, the capital city, represents 32% (642,410/1,987,093) of the population.

### Case Definitions

IPD case in the study was defined according to EU case definition, which was established in 2012 (15). In cases where *S. pneumoniae* was detected in blood and cerebrospinal fluid (CSF) simultaneously, cases were classified as meningitis. All IPD diagnosis for surveillance data were coded according to ICD 10 5-th version: B95.3 and A40.3 for septicaemia, and G00.1 and G00.2 for meningitis.

### IPD Surveillance in Latvia

Laboratory confirmed IPD cases (diagnosis B95.3, A40.3, G00.1, G00.2) are reported to the Center for Disease Prevention and Control of Latvia (CDPC) by clinicians and all *S. pneumoniae* positive isolates from normally sterile sites should be reported to the CDPC by laboratories (13). According to the European Union case definitions (15) and cabinet regulations (13), clinicians should report to the CDPC in writing within three working days by sending a completed urgent report form by fax, by post, by courier, or electronically, and register the fact of notification in the medical documentation of the patient. Laboratories, according to the same regulations, should report all positive *S. pneumoniae* isolates from normally sterile sites to the CDPC in the same way. Clinicians using the urgent report form provide information about patient's initial diagnosis, personal data, laboratory findings, and vaccination status if applicable.

### Immunization Coverage Estimates

Immunization coverage was estimated for PCV10 first, second, and third doses. For numerator we used number of first, second, and third PCV10 doses distributed during the study period and documented according to Cabinet regulations (16). Denominators were the population of target children.

### Laboratory Methods

#### Identification

Identification was performed using conventional methods or an automated system, validated in the National Reference laboratory. Swabs were streaked onto tryptic soy agar plates supplemented with 5% sheep blood and incubated aerobically at 36±1°C 24–48 h in 5% CO_2_-enriched air. Suspected haemolytic colonies were chosen for identification, colored by Gram, and tested for inhibition by optochin. Identification was performed in the VITEK^®^ 2 COMPACT.

#### Serotyping

Two methods were used to determine serotypes: latex agglutination and Quellung test [ImmuLexTMPneumotest; Quellung reaction (SSI^®^Pneumotest)] (Statens Serum Institut, Denmark). All serotypes that were not possible to identify were classified as non-typeable.

### Statistical Analysis

We generated two null hypotheses: (a) no trends in *S. pneumoniae* serotypes replacement during the study period and (b) no association between *S. pneumoniae* serotypes and case fatality.

We calculated IPD incidence by time, age, and sex. We calculated IPD case fatality and trends in *S. pneumoniae* serotypes. Trends in serotypes, age, sex, and outcome were assessed with chi2 test for the odds trend. Population estimation was provided by the Central Statistical Bureau of Latvia (14).

*S. pneumonia* serotypes detected in IPD cases were grouped by vaccine constituent serotypes, clinical presentation, sex, and age. We conducted univariable analysis to identify risk factors associated with death from IPD using attack rate (AR), relative risks (RR), and 95% confidence interval (95%CI) for each risk factor.

We conducted backwards logistic regression to develop the multivariable model, using risk factors that had *p* < = 0.2 during univariable analysis. Removing variables, with the highest *p*-values, step-by-step we used Bayesian information criterion (BIC) test to compare different models. We checked for confounders using a change of 20% in the other risk factors as a cut off. We chose the final model with the lowest BIC test results. Univariable and multivariable data analysis were performed using STATA v12 (Stata Corporation, Texas, USA).

*S. pneumoniae* serotypes were grouped by vaccine constituent serotypes: PCV10, PCV13nonPCV10, PPV23nonPCV13, and Non-Vaccine serotypes (Table 1).

**Table 1:** *S. pneumoniae* serotype groups according vaccine constituent serotypes.

***S. pneumoniae* serotype**	***S. pneumoniae* serotyps**
**groups**	
PCV10	1, 4, 5, 6B, 7F 9V, 14, 18C, 19F, 23F
PCV13non10	3, 19A, 6A
PPV23nonPCV13	8, 9N, 10A, 11A, 12F, 15B, 17F, 20, 22F
Non-Vaccine	Any serotypes not in PCV13 and not in PPV23

## Results

### IPD Cases and Incidence From Surveillance Data

Over the 7-year period (2012–2018) 466 cases of IPD were reported to the CDPC of Latvia. The notified incidence remained stable from 2012 to 2014 (2.7 per 100 000 population), peaked in 2015 (4.4 per 100 000 population), and fell to 3.9 per 100 000 population in 2018.

**Figure 1:**
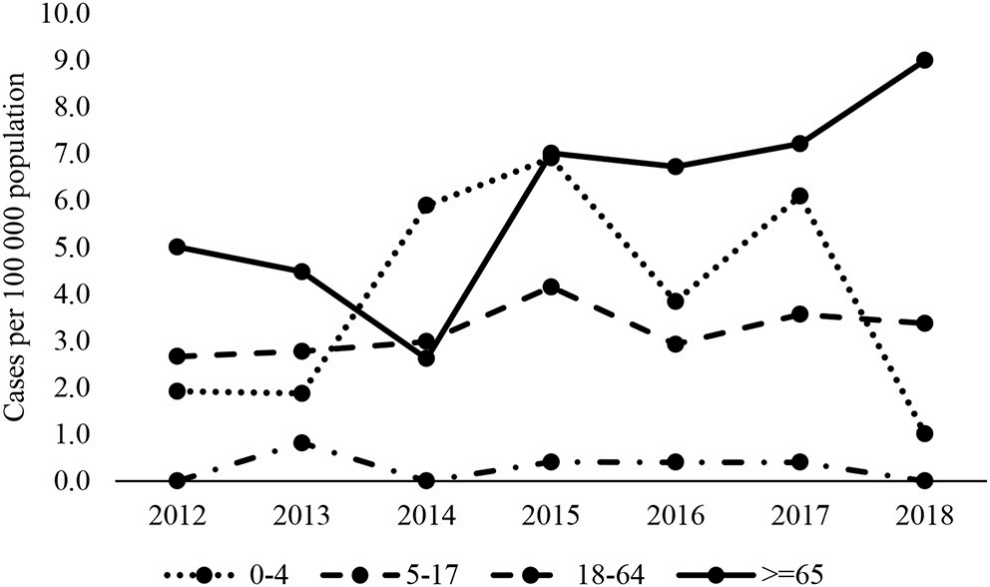
Trends in IPD incidence, distributed by age groups, 2012–2018.

Over the period, the highest mean annual IPD incidence was in infants aged under 1 year, at 4.8 per 100 000 population, and in the elderly aged 65 and older, at 6 per 100 000 population (Table 2). Over the period of surveillance, incidence rates in children under 4 appeared to fall in 2018 but rose in adults aged 65 and over from 2015 onwards (Figure 1). Rates in adults under 65 years remained consistent.

**Table 2:** Number of IPD cases and average annual IPD incidence by age groups, 2012–2018.

	**Number of cases**	**Average annual incidence**	**95%CI**	**Number of cases in male (%)**
<1	7	4.8	1.2–26.9	3 (43%)
1–4	12	2	2.9–8.7	8 (67)
5–14	5	0.4	1.3–2.8	4 (80)
15–64	280	3.1	2.2–4.2	201 (72)
≥65	162	6	3.8–9	68 (42)
Total	466	3.4	2.6–4.2	284 (61)

Of the IPD cases, 61% (284/422) were detected in males; the highest mean annual incidence was reported in males (4.5 per 100 000 inhabitants) in comparison to females (2.4 per 100 000 inhabitants) (Incidence ratio-1.8 CI 1.6–2.4). In all age groups, the highest incidence was in males, except for in infants aged under one where the highest mean annual incidence was detected in females (5,7 cases per 100 0000 inhabitants). The peak year in both sexes was 2015: 6 per 100 000 in males and 3 per 100 000 in females.

Septicaemia (diagnoses B95.3, A40.3) represents 77% (358/466) of IPD cases. 23% (106/466) of IPD cases were defined as meningitis and 0.4% (2/466) of *S. pneumoniae* isolated were detected from other sterile sites.

### Serotypes

Over the 7-year period, 90% (421/466) of samples were serotyped. The proportion of samples with serotyping increased from 71% (40/56) in 2012 to 97% (74/76) in 2018 (chi2 test for trend of odds = 0.000).

**Figure 2:**
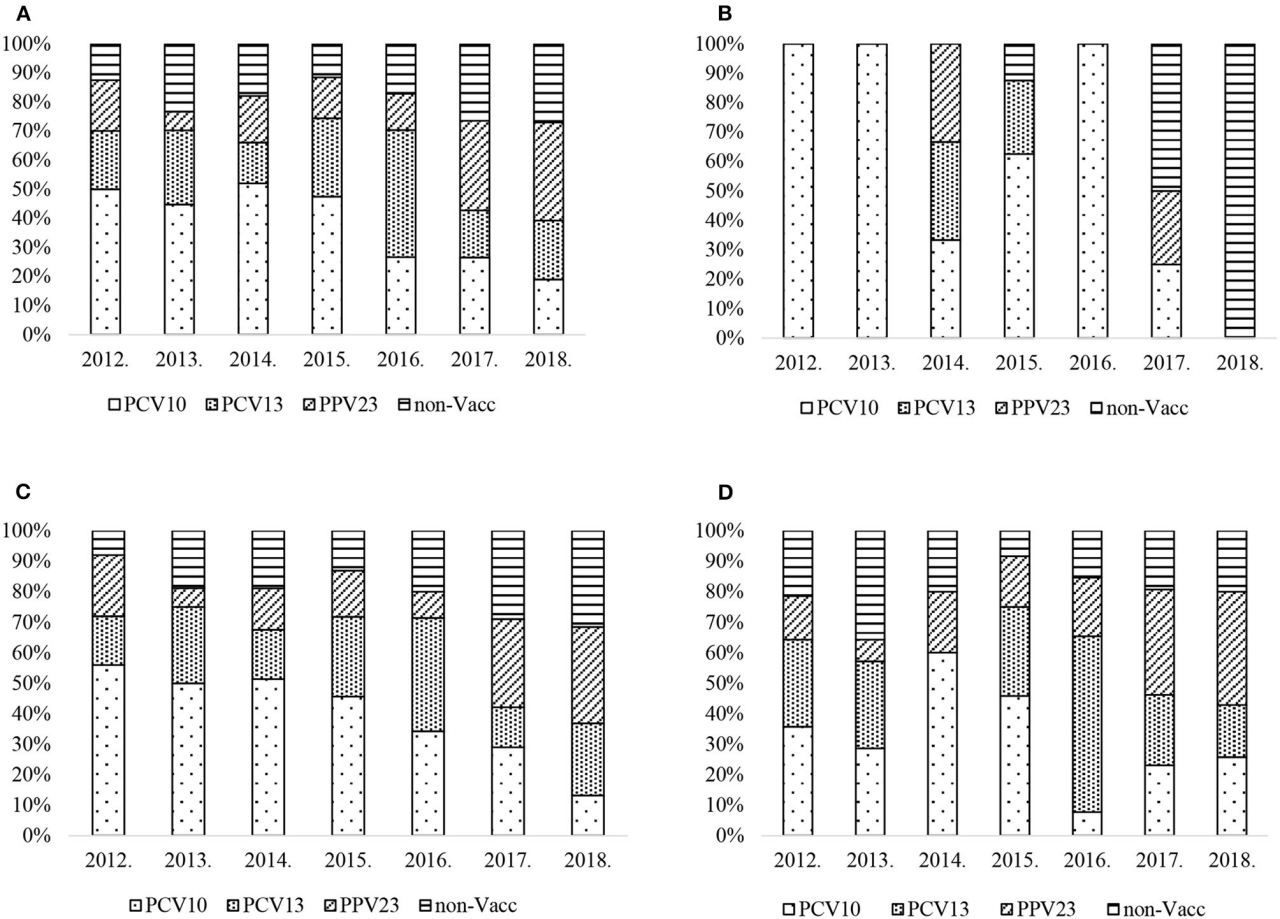
**(A)** *S. pneumoniae* serotype percentage by year and PCV vaccine type, all age groups. **(B)** *S. pneumoniae* serotype percentage by year and PCV vaccine type, age group <18. **(C)** *S. pneumoniae* serotype percentage by year and PCV vaccine type, age group 18–64. **(D)** *S. pneumoniae* serotype percentage by year and PCV vaccine type, age group ≥65.

Although PCV10 vaccine serotypes were the most prevalent in IPD cases up to 2015 (Figure 2A), there was a decreasing trend from 50% (20/40) in 2012 to 19% (14/74) in 2018 (chi2 test for trend of odds = 0.000).

Specific serotypes found in PCV13nonPCV10 showed particular trends: serotype 3 fell from 17% (7/40) in 2012 to 8% (6/74) in 2018 with a peak of 29% (19/64) in 2016 (chi2 test for trend of odds *p* = 0.02). Serotype 19A had an increasing trend, rising from 2% (1/50) in 2014 to 12% (9/74) in 2018 (chi2 test for trend of odds *p* = 0.000). From 2017, PPV23nonPCV13 and Non-Vaccine serotypes become more common. The PPV23nonPCV13 vaccine serotypes have an increasing trend and rose from 18% (7/40) to 34% (25/74) (chi2 test for trend of odds = 0.000). Non-Vaccine serotypes have an increasing trend and rose from 13% (5/40) to 27% (20/74) (chi2 test for trend of odds = 0.038).

In the age group <18 there was no trend observed in all serotype groups (Figure 2B). During the study period there were only 24 IPD cases reported in this age group.

PCV10 serotypes had a decreasing trend in the age group 18–64 (Figure 2C) and fell from 56% (14/25) to 13% (5/38) (chi2 test for trend of odds = 0.000). There was no trend observed in PCV13nonPCV10 serotype group, which peaked at 37% (13/35) in 2016, however serotype 19A 11% (5/46) was reported in 2015, then fell to 6% (2/35) in 2016 and increased to 11% (4/38) in 2018 (chi2 test for trend of odds = 0.012). No trend was observed in serotype 3. PPV23nonPCV13 serotype group has an increasing trend and rose from 9% (3/35) in 2012 to the highest proportion at 32% (12/38) in 2018 (chi2 test for trend of odds = 0.014), whereas Non-Vaccine serotypes rose from 8% (2/25) to 32% (12/38) (chi2 test for trend of odds = 0.013) in the period.

In the age group 65 and over (Figure 2D), no trend was observed in PCV10, PCV13nonPCV10, or Non-Vaccine serotypes collectively. However, serotype 3 fell from 29% (4/14) in 2012 to 3% (1/35) in 2018 with a peak of 34% (9/26) in 2016 (chi2 test for trend of odds = 0.017). Serotype 19A peaked in 2016 at 23% (6/26), and overall had an increased trend (8% (2/24) in 2015 to 14% (5/35) in 2018 (chi2 test for trend of odds = 0.023). PPV23nonPCV13 serotype group had an increasing trend and rose from 14% (2/14) to 37% (13/35) (chi2 test for trend of odds = 0.000).

### Case Fatality

Total case fatality during the study period was 19% (87/466) with no trend over the period. The highest case fatality at 36% (20/56) was reported in 2013.

By age, the highest total case fatality was 23% reported in the age group 65 years and over (37/162), with a similar peak in 2013 of 47% (8/17).

There were no case fatalities reported in age group <18.

The highest case fatality risk was associated with serotypes in PCV13nonPCV10 (30.1%) and constituent serotype 3 (30.8%) and serotype 19A (28.1%). In univariable analysis, factors significantly associated with IPD case fatality were PCV13nonPCV10 serotypes with crude case fatality risk ratio 2.04 (95%Confidence interval (CI) 1.37–3.02) and *S. pneumoniae* serotype 3 with crude case fatality risk ratio 1.91 (95% CI 1.25–2.93). Although serotype 19A did not present significant associations with lethal outcome, it still has a high case fatality risk ratio 1.59 (95% CI 0.88–2.87) (Table 3).

**Table 3:** Factors associated with IPD case fatality.

**Factor**	**Deaths/cases**	**Case fatality Risk % (CFR)**	**Case fatality risk ratio (RR)**	**95%CI of RR**	***p*-value of RR**
PCV10 serotype	15/153	9.8	0.42	0.25–2.71	0.000
PCV13nonPCV10 serotype	31/103	30.1	2.04	1.37–3.02	0.001
Serotype 3	21/68	30.8	1.91	1.25–2.93	0.004
Serotype 19A	9/32	28.1	1.59	0.88–2.87	0.146
Non-Vaccine	19/82	23.2	1.33	0.84–2.10	0.228
PPV23nonPCV13 serotype	13/83	15.7	0.81	0.47–1.40	0.453
Male sex	58/284	20.4	1.28	0.85–1.92	0.225
Septicaemia	67/356	18.8	1.04	0.66–1.63	0.881
Meningitis	20/108	18.5	0.99	0.63–1.55	0.963
Age^*^			1.02 (OR^**^)	1.00–1.03	0.002

* *Continuous variable*.

** *Odds ratio*.

Conducting multivariable analysis with logistic regression, age [adjusted odds ratio (OR) 1.01 and 95% –CI 1.0–1.03 *p* = 0.006] and *S. pneumoniae* serotype 3 (adjusted OR 2.3 95%CI 1.25–4.12 *p* = 0.007) were independent and significant associations with death from IPD. Alternatively, using PCV13 serotypes in the logistic regression model, instead of serotype 3, and adjusting for age, we found independent and significant association with death (adjusted OR 2.3 95%CI 1.36–3.93 *p* = 0.002) (Table 3).

### Vaccination Coverage Against Pneumococcal Infection in Latvia

Since PCV10 was introduced into the national immunization program, the coverage of the first PCV10 dose commenced at 82.6% in 2012, stabilizing at 93% from 2016 onwards (Table 4). In 2018, 93.3% (the 1st dose) of children started their vaccination against pneumococcal infection but only 84% completed their vaccination (3rd dose), so the gap observed between the 1st and 3rd dose is 9.3%.Vaccination coverage data in the adult population is not available.

**Table 4:** Vaccination coverage (%) with PCV10, 2012–2018.

	**2012**	**2013**	**2014**	**2015**	**2016**	**2017**	**2018**
PCV1	82.6	86.9	85.7	89.6	92.4	92.1	93.3
PCV2	81.5	91.1	85.9	89.1	92.5	92.1	92.5
PCV3	55.5	86.9	83.8	85.1	84.3	86.6	84.0

## Discussion

The overall proportion of PCV10 serotypes indicates a significantly decreasing trend but there is a significant increasing trend in serotype 19A and decreasing trend in serotype 3 in all age groups. This is similar to the pediatric population in Finland, where there is a decreasing trend in PCV10 serotypes in children aged <5 years representing only 19 % of all IPD isolates reported in 2014. Moreover, serotype 19A has had an increasing trend in children since 2010 when PCV10 was introduced to the immunization program and by year 2015, serotype 19A was detected in half the total IPD cases (17). The most common serotypes in Latvia, according to study data, in adult populations in 2014 were 3 (17 %), 22F (12 %), and 19A (11 %). Serotype 19A also became prevalent in older age groups in the Netherlands and Austria (8, 18). The same trend was observed in the Czech Republic and Slovakia, where in 2012–2013 serotype 19A was frequently detected in the pediatric population (19). Serotype 19A also has an increasing trend in both <5 and ≥5 in Brazil (since 2010) and in Chile (since 2011) (17).

PPV23nonPCV13 vaccine serotypes have an increasing trend overall and in all age groups. The same is observed with Non-Vaccine serotypes, except in age group ≥65. These trends are possibly responsible for the rising incidence age group ≥65. SpidNet report that the same trend in older adults was reported from other PCV10/13 European countries, suggesting serotype replacement and, considering indirect immunization effects, questioning the immunization program benefits on IPD in elderly (20).

Multivariable analysis indicated serotype 3 and age were independently and significantly associated with IPD case fatality. Moreover, our study indicated significant association with PCV13nonPCV10 serotypes. This may relate to serotype 19A which was also associated with death, but was not significant due to the small number of cases reported. Thus, the introduction of vaccinations covering more *S. pneumoniae* serotypes could decrease IPD case fatality as well as burden of disease. IPD surveillance data do not provide data about IPD patients' comorbidities, treatment, and other additional information about risk factors.

During the last 7 years, IPD notification rate varied from 2.5 in 2014 to 4.4 in 2015. The elderly and infants are the most affected age groups, similar to other European countries (21). A Canadian study also reports higher IPD incidence in individuals aged ≥75 then those aged 17–54 (22). In the Baltic states, the latest updates from the ECDC surveillance Atlas of Infectious Diseases indicates that during the study period Latvia has the highest IPD incidence in comparison with its neighboring countries, Estonia and Lithuania. Estonia reports 3.42 and Lithuania 2.67, while Latvia had 3.9 cases per 100 000 population in 2017.

Latvia saw similar serotypes to other European countries: 3, 19A, 9N, and 8. However, serotypes 12F, 22F, 15A, 10A, 33F, and 11A were common in Europe in 2016 but uncommon in Latvia (21). We found that the most common serotypes during the study period were 4, 19F, 7F, 6B, 9V, and 14. Non-Vaccine serotypes (8, 10A, 12F, 24F) were reported in children aged <4 years in 2016 (21).

Overall, the IPD case fatality in Latvia from 2012 to 2018 was 19%, with the highest risk in the older age group. Similar trends are reported by the ECDC and Canadian study where reported case fatality in age group ≥75 was 32 and 10%, respectively, for those aged 17–54 (21, 22). There are a lot of countries in the EU which have recommended vaccination against pneumococcal disease for adults aged 65+, but are not funded by the government. CRF in the age group 65+ varies in these countries. A descending trend is found in CFR for age group 65+ in some countries, such as Hungary from 54% in 2015 to 29.3% in 2018, Greece from 25% in 2014 to 20% in 2018, Portugal from 20.8% in 2015 to 17.8% in 2018, and Norway from 15.4% in 2014 to 10.8% in 2018. However, in other countries the CFR in age group 65+ shows increasing trends or no trend despite recommended vaccination against pneumococcal disease (Iceland from 17.6% in 2014 to 30.8% in 2018, the UK from 8.2% in 2014 to 23.2% in 2018, Italy from 4.2% in 2015 to 20% in 2018, Ireland from 4.5% in 2014 to 18.6% in 2018, and Slovenia from 6.9% in 2014 to 14.3% in 2018).

National health service data provided in 2018 reported the total number of hospitalized patients with a discharge diagnosis of pneumonia due to *Streptococcus pneumoniae (J13)* was 106, that is 30 cases more then all IPD cases reported via the national surveillance system in 2018 (76 IPD cases). Although a diagnosis of pneumonia does not imply sepsis, this is still suggestive of under-reporting to the surveillance system. It is likely that some cases of pneumonia would have had sepsis as well, and thus should have been notified. Under-reporting could also occur if blood samples were not taken or antibiotics were used before the blood cultures were taken or had inadequate samples (e.g., insufficient blood volume) to define sepsis with blood cultures (23).

Underreporting in a surveillance system and possible under-diagnosis are likely to be study limitations, although our notification rates are comparable or higher than other Baltic states (21). Moreover, there are not clear guidelines for hospitals' laboratories concerning sample logistics. Some hospitals, when collecting samples, conduct *S. pneumoniae* identification first in their own laboratories, then send *S. pneumoniae* isolates to National Reference laboratory (NRL) for further serotyping. There is no information about identification tests' validation in regional hospitals' laboratories. No PCR has been performed in regional hospitals' laboratories. Some hospitals send blood or CSF or other sterile sites for further *S. pneumoniae* identification and serotyping directly to the NRL, which chose *S. pneumoniae* isolation but not PCR, so we proposed that sample underestimation of cases could have occurred. Thus, we suggest evaluating the IPD surveillance system for completeness and representativeness of surveillance data.

Our study results, particularly the increasing trend of 19A serotype, could lead to the national advisory committee of immunization to consider changing to a higher valent pneumococcal conjugant vaccine. Moreover, Latvia does not have any vaccine recommendations for the elderly yet, but our results provide information that the IPD incidence, particularly in elderly, is increasing and age is a significant risk factor associated with death.

## Conclusions

The main null hypothesis is rejected as our data indicate evidence of serotype replacement with an increasing trend of serotype 19A and PPV23nonPCV13, Non-Vaccine serotypes.

Our study demonstrated serotype 3 and age as independent and significant factors associated with fatal IPD outcome; thus, the second null hypothesis is also rejected.

Further studies of *S. pneumoniae* carriage would be useful in providing more evidence to characterize serotypes' circulation.

## Data Availability Statement

The datasets generated for this study are available on request to the corresponding author.

## Ethics Statement

This study was held on IPD surveillance data analysis without any personal data. So we could not identify any person whom to require written informed consent. Therefore, ethical approval for this study and written informed consent from the participants of the study were not required in accordance with local legislation and national guidelines.

## Author Contributions

LS and SB conceived of the presented idea. LS wrote the manuscript. IZ and AV contributed to material and methods section. ED and SB contributed to result section. AK, IG, LV, and HC contributed to the discussion section. HC additional data collection from National children's hospital, revised, and approved the final version. All authors contributed to manuscript revision and read and approved the final version.

## Funding

This work was supported by the Latvijas Infektologu un Hepatologu asociācija.

## Conflict of Interest

The authors declare that the research was conducted in the absence of any commercial or financial relationships that could be construed as a potential conflict of interest.

## Publisher's Note

All claims expressed in this article are solely those of the authors and do not necessarily represent those of their affiliated organizations, or those of the publisher, the editors and the reviewers. Any product that may be evaluated in this article, or claim that may be made by its manufacturer, is not guaranteed or endorsed by the publisher.
